# Supplementary figures and images for: Allocating epidemic response teams and vaccine deliveries by drone in generic network structures, according to expected prevented exposures
Source: PLoS One. 2021 Mar 5;16(3):e0248053. doi: 10.1371/journal.pone.0248053 (PMC7935281; doi:10.1371/journal.pone.0248053)

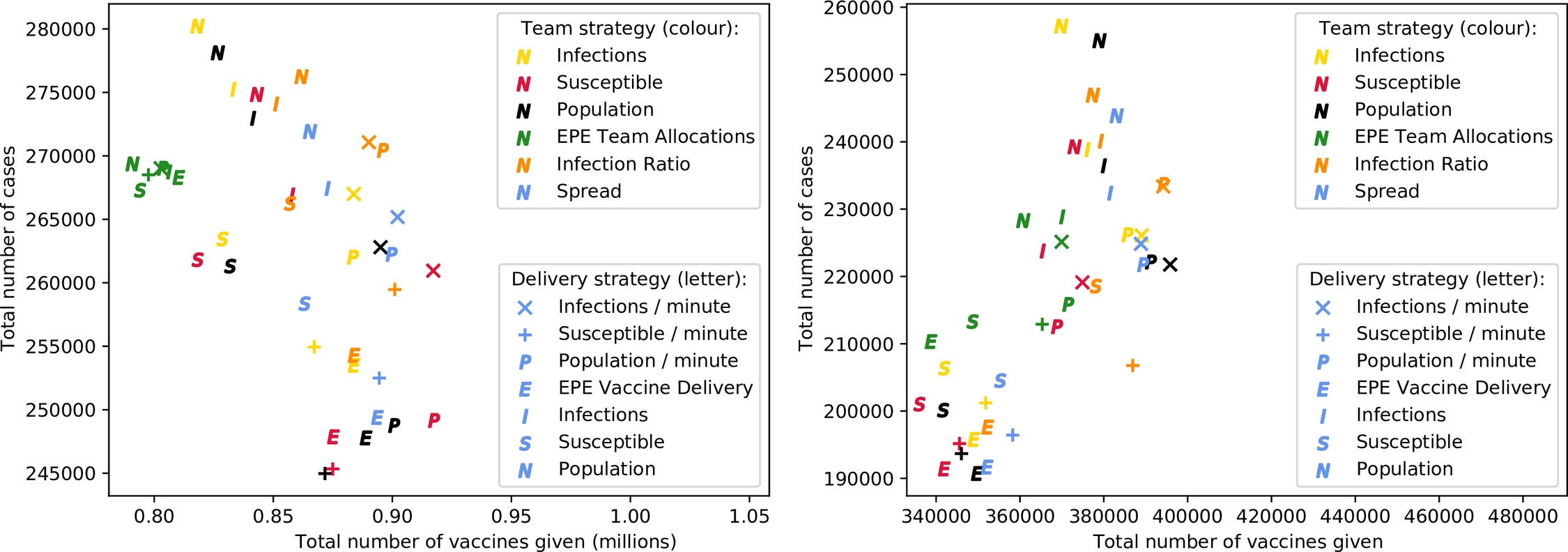

Supplement: S1 Fig — Results for the untargeted intervention are depicted on the left, and results for the targeted intervention are on the right. (TIF) [file pone.0248053.s001.tif]

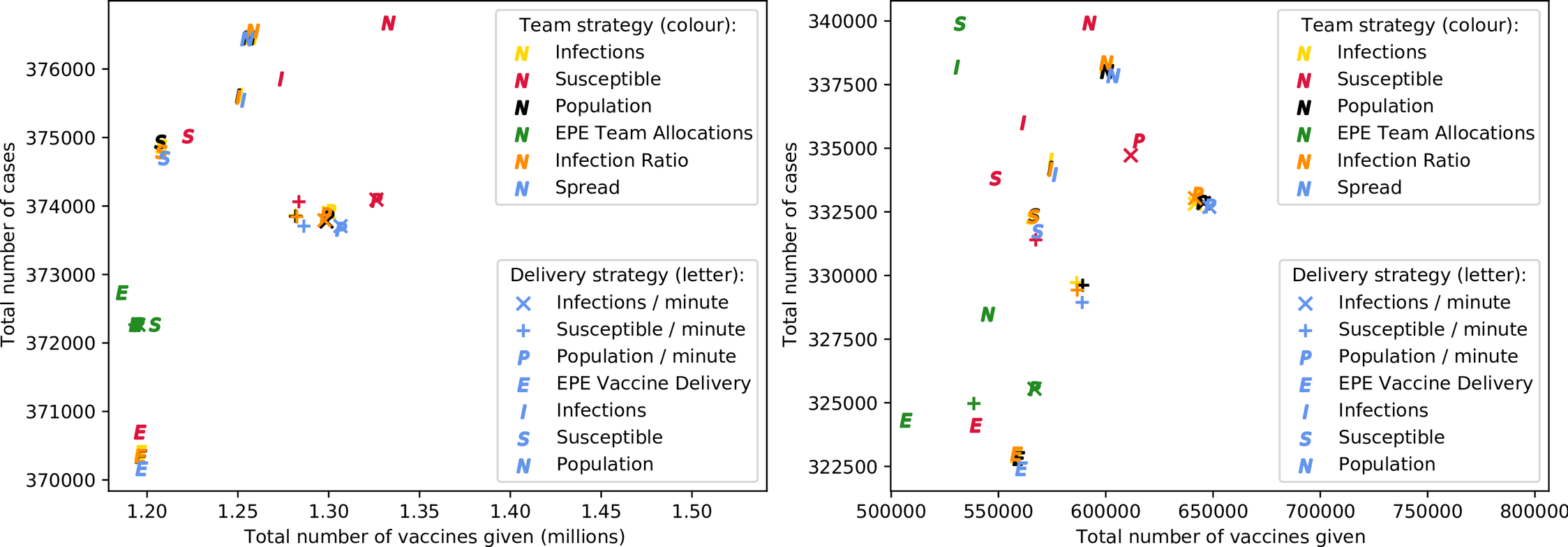

Supplement: S2 Fig — Results for the untargeted intervention are depicted on the left, and results for the targeted intervention are on the right. (TIF) [file pone.0248053.s002.tif]

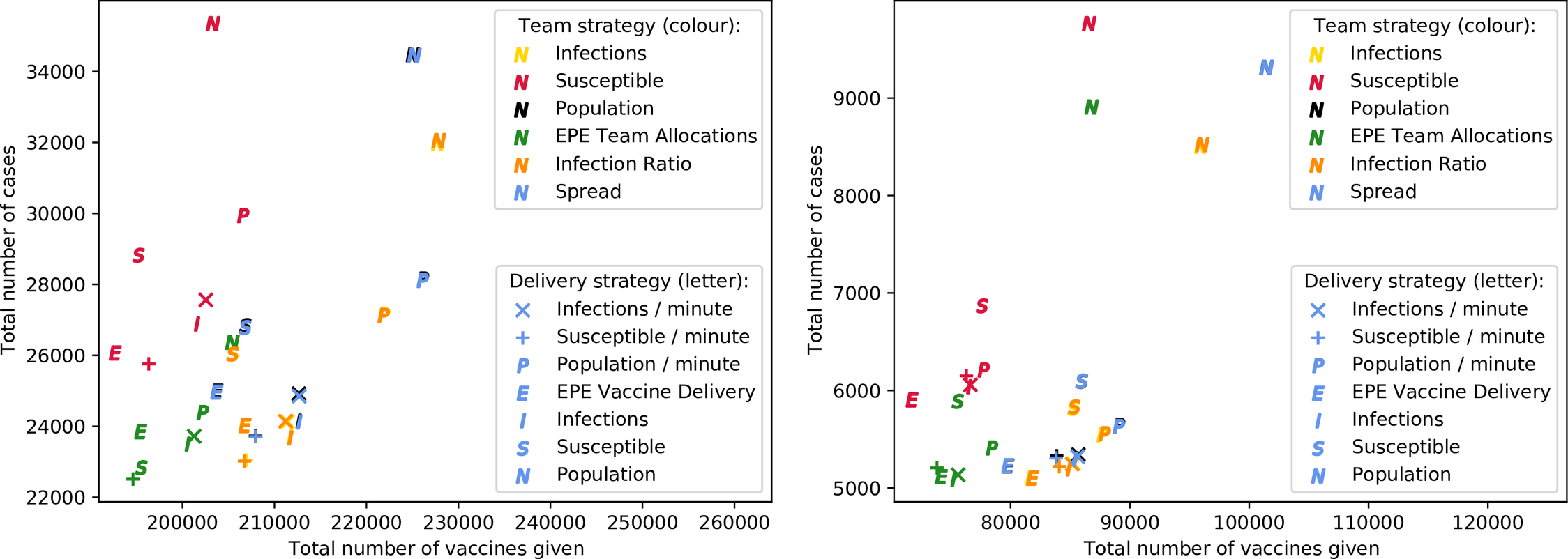

Supplement: S3 Fig — Results for the untargeted intervention are depicted on the left, and results for the targeted intervention are on the right. (TIF) [file pone.0248053.s003.tif]

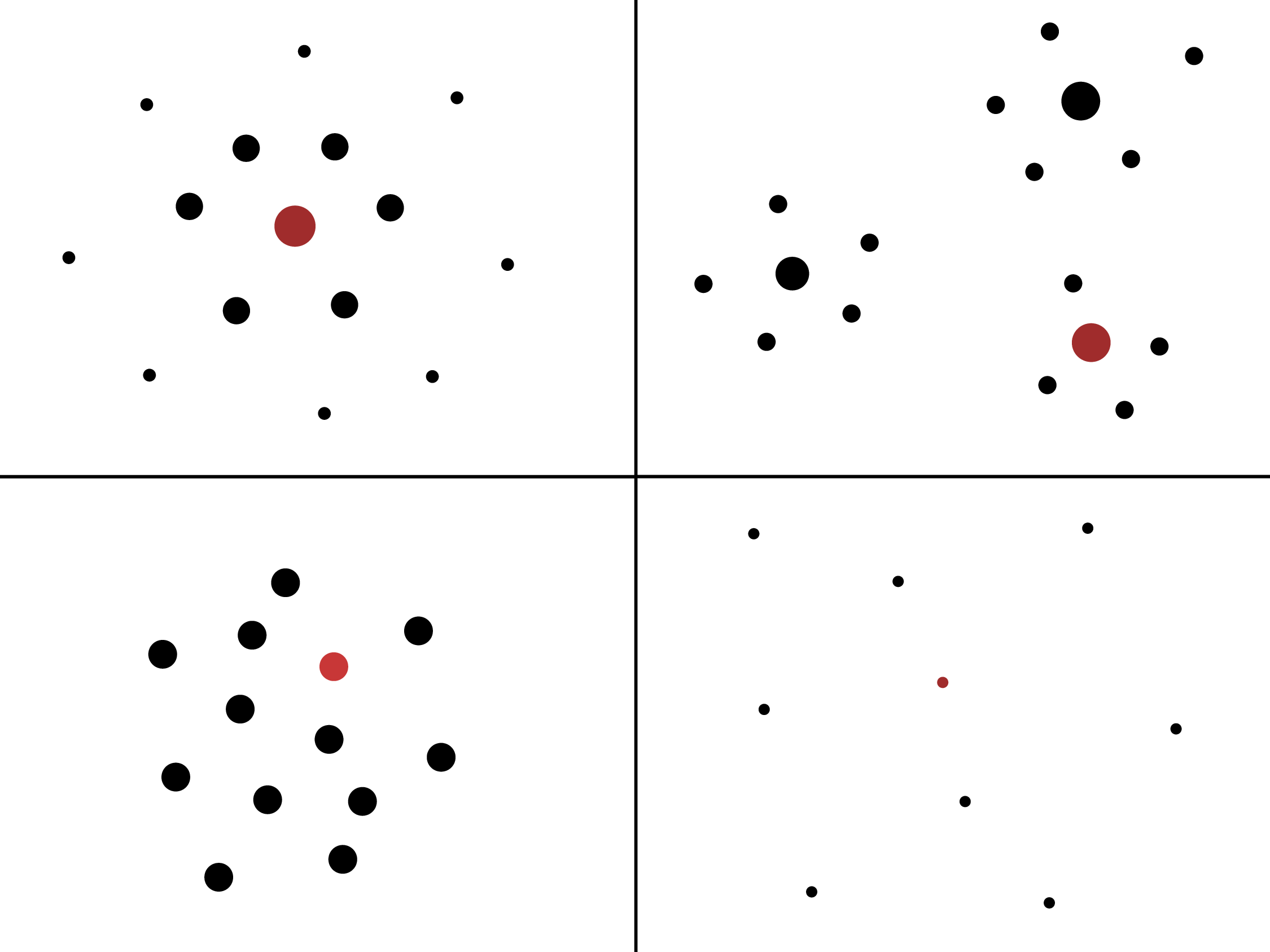

Supplement: S4 Fig — The population size at each network location is represented by the radius of the dot, and the epicentre is marked in red. The monocentric network is top left, the polycentric network is top right, the city-type network is bottom left, and the rural-type network is bottom right. (TIF) [file pone.0248053.s004.tif]

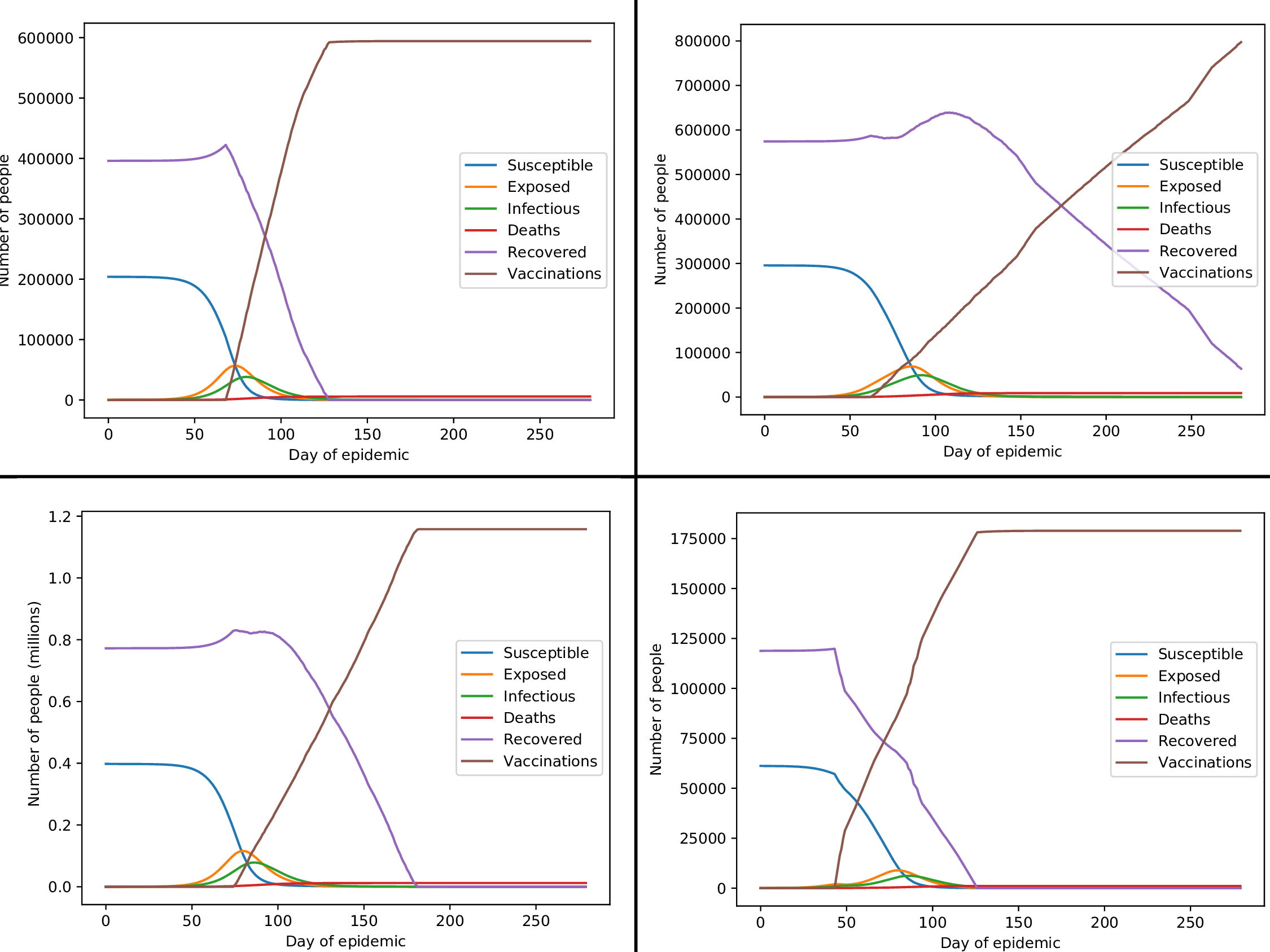

Supplement: S5 Fig — The below plots depict the progression of a single simulated measles epidemic, in each of the four network types considered. Each of the four simulations were performed with the default set of parameters, and untargeted vaccination. The plots each give an indication of how the total network population progresses between the S, E, I, R, V, and D categories in that network. The monocentric network is top left, the polycentric network is top right, the city-type network is bottom left, and the rural-type network is bottom right. (TIF) [file pone.0248053.s005.tif]
