# Supplementary material for: Allocating epidemic response teams and vaccine deliveries by drone in generic network structures, according to expected prevented exposures
Source: PLoS One. 2021 Mar 5;16(3):e0248053. doi: 10.1371/journal.pone.0248053 (PMC7935281; doi:10.1371/journal.pone.0248053)
Supplement: S1 Table — (PDF) [file pone.0248053.s006.pdf]

S1 Table. Input dataset for monocentric network structure.

Location	x	y	Population	Index E	Index I
Centre	198	148	100000	0	10
Inner 1	175	195	50000	0	0
Inner 2	224	196	50000	0	0
Inner 3	254	163	50000	0	0
Inner 4	229	109	50000	0	0
Inner 5	169	97	50000	0	0
Inner 6	143	163	50000	0	0
Outer 1	211	253	25000	0	0
Outer 2	295	227	25000	0	0
Outer 3	323	135	25000	0	0
Outer 4	281	73	25000	0	0
Outer 5	222	53	25000	0	0
Outer 6	125	74	25000	0	0
Outer 7	81	139	25000	0	0
Outer 8	124	224	25000	0	0
