# Supplementary material for: Allocating epidemic response teams and vaccine deliveries by drone in generic network structures, according to expected prevented exposures
Source: PLoS One. 2021 Mar 5;16(3):e0248053. doi: 10.1371/journal.pone.0248053 (PMC7935281; doi:10.1371/journal.pone.0248053)
Supplement: S2 Table — (PDF) [file pone.0248053.s007.pdf]

S2 Table. Input dataset for polycentric network structure.

Location	x	y	Population	Index E	Index I
Cluster 1, Centre	85	109	150000	0	0
Cluster 1, Location 2	81	155	30000	0	0
Cluster 1, Location 3	135	132	30000	0	0
Cluster 1, Location 4	125	91	30000	0	0
Cluster 1, Location 5	75	74	30000	0	0
Cluster 1, Location 6	38	108	30000	0	0
Cluster 2, Centre	259	67	150000	0	10
Cluster 2, Location 2	254	108	30000	0	0
Cluster 2, Location 3	305	71	30000	0	0
Cluster 2, Location 4	285	34	30000	0	0
Cluster 2, Location 5	240	48	30000	0	0
Cluster 3, Centre	253	209	150000	0	0
Cluster 3, Location 2	241	256	30000	0	0
Cluster 3, Location 3	326	242	30000	0	0
Cluster 3, Location 4	289	181	30000	0	0
Cluster 3, Location 5	232	173	30000	0	0
Cluster 3, Location 6	209	213	30000	0	0
