# Supplementary material for: Allocating epidemic response teams and vaccine deliveries by drone in generic network structures, according to expected prevented exposures
Source: PLoS One. 2021 Mar 5;16(3):e0248053. doi: 10.1371/journal.pone.0248053 (PMC7935281; doi:10.1371/journal.pone.0248053)
Supplement: S3 Table — (PDF) [file pone.0248053.s008.pdf]

S3 Table. Input dataset for city network structure.

Location	x	y	Population	Index E	Index I
1	192	246	90000	0	0
2	171	213	90000	0	0
3	115	201	90000	0	0
4	277	215	90000	0	0
5	223	193	90000	0	10
6	164	166	90000	0	0
7	220	147	90000	0	0
8	123	123	90000	0	0
9	291	135	90000	0	0
10	181	108	90000	0	0
11	241	107	90000	0	0
12	150	59	90000	0	0
13	229	71	90000	0	0
