# Supplementary material for: Allocating epidemic response teams and vaccine deliveries by drone in generic network structures, according to expected prevented exposures
Source: PLoS One. 2021 Mar 5;16(3):e0248053. doi: 10.1371/journal.pone.0248053 (PMC7935281; doi:10.1371/journal.pone.0248053)
Supplement: S4 Table — (PDF) [file pone.0248053.s009.pdf]

S4 Table. Input dataset for rural network structure.

Location	x	y	Population	Index E	Index I
1	62	268	20000	0	0
2	155	238	20000	0	0
3	277	272	20000	0	0
4	69	155	20000	0	0
5	184	173	20000	0	10
6	334	143	20000	0	0
7	199	96	20000	0	0
8	100	38	20000	0	0
9	253	31	20000	0	0
