# Supplementary material for: Allocating epidemic response teams and vaccine deliveries by drone in generic network structures, according to expected prevented exposures
Source: PLoS One. 2021 Mar 5;16(3):e0248053. doi: 10.1371/journal.pone.0248053 (PMC7935281; doi:10.1371/journal.pone.0248053)
Supplement: S5 Table — (PDF) [file pone.0248053.s010.pdf]

S5 Table. Input dataset for Niamey epidemic validation.

Location	x	y	Population	Index E	Index I
Centre	198	148	20000	0	10
Inner 1	175	195	14000	0	0
Inner 2	224	196	14000	0	0
Inner 3	254	163	14000	0	0
Inner 4	229	109	14000	0	0
Inner 5	169	97	14000	0	0
Inner 6	143	163	14000	0	0
Outer 1	211	253	7000	0	0
Outer 2	295	227	7000	0	0
Outer 3	323	135	7000	0	0
Outer 4	281	73	7000	0	0
Outer 5	222	53	7000	0	0
Outer 6	125	74	7000	0	0
Outer 7	81	139	7000	0	0
Outer 8	124	224	7000	0	0
